# Comparative Studies on Polyurethane Composites Filled with Polyaniline and Graphene for DLP-Type 3D Printing

**DOI:** 10.3390/polym12010067

**Published:** 2020-01-02

**Authors:** Hyeonseo Joo, Sunghun Cho

**Affiliations:** School of Chemical Engineering, Yeungnam University, Gyeongsan 38541, Korea; hyeonssjoo@gmail.com

**Keywords:** 3D printing, digital light processing, polyurethane, polyaniline, graphene

## Abstract

Digital light processing (DLP)-type 3D printing ensures several advantages, such as an easy solution process, a short printing time, high-quality printing, and selective light curing. Furthermore, polyurethane (PU) is among the promising candidates for 3D printing because of its wide range of applications. This work reports comparative studies on the fabrication and optimization of PU composites using a polyaniline (PANI) nanomaterial and a graphene sheet (GS) for DLP-type 3D printing. The morphologies and dispersion of the printed PU composites were studied by field emission scanning electron microscope (FE-SEM) images. Bonding structures in the PU composites were investigated by Fourier-transform infrared (FT-IR) spectroscopy. As-prepared PU/PANI and PU/GS composites with different filler contents were successfully printed into sculptures with different sizes and shapes. The PU/PANI and PU/GS composites exhibit the improved sheet resistance, which is up to 8.57 × 10^4^ times (1.19 × 10^6^ ohm/sq) lower and 1.27 × 10^5^ times (8.05 × 10^5^ ohm/sq) lower, respectively, than the pristine PU (1.02 × 10^11^ ohm/sq). Moreover, the PU/PANI and PU/GS composites demonstrate 1.41 times (44.5 MPa) higher and 2.19 times (69.3 MPa) higher tensile strengths compared with the pristine PU (31.6 MPa). This work suggests the potential uses of highly conductive PU composites for DLP-type 3D printing.

## 1. Introduction

Three-dimensional printing is among the future-oriented manufacturing technologies that can significantly contribute to the fourth industrial revolution, and 3D printing makes it easy to create custom products with the desired designs, shapes, and sizes for various applications, such as machinery, jewelry, automobile, dental, electronic products, medicine, tissue engineering, construction materials, and so forth [1,2,3]. There are various technologies involved in 3D printing, such as material extrusion, stereolithography (SLA), digital light processing (DLP), powder bed fusion, material jetting, binder jetting, and powder jet fusion [1,2,3]. Although material extrusion technology can be low-cost and provide a simple process for 3D printing, the resolution of the printed sculptures is low, and a long processing time is required to conduct material extrusion. SLA provides a high vertical resolution and a high printing quality, but the SLA method also requires a long processing time [1,2,3]. DLP-type 3D printing provides both shorter printing times and a superior printing quality compared with material extrusion. In particular, DLP-type 3D printing allows high resolutions in the X–Y plane and Z-axis [4,5,6,7,8,9,10,11,12,13,14,15,16,17,18]. A variety of polymers, including acrylonitrile-butadiene-styrene (ABS) resin [1], silicone rubber [4], acrylic resin [5,6,7], polycaprolactone (PCL) [8], polyimide (PI) [9], polyvinylpyrrolidone (PVP) [10], and polyurethane (PU) [11,12], were used as materials for 3D printing. Stretchable silicone elastomer, with 1100% of strain at a break point, was realized by the DLP-type 3D printing of silicone prepolymers [4]. Among acrylic resins, poly(ethylene glycol) diacrylate (PEGDA) has been the most widely used material for DLP-type 3D printing [6,7]. Zarek et al. reported the DLP-type 3D printing of polycaprolactone (PCL) for use as a shape memory device [8].

In particular, various advantages of PU have attracted a great deal of interest in its application to DLP-type 3D printing [11,12]. Polyurethane (PU) and its composites are thermosetting polymers, meaning that the PU-based composites can exhibit the properties of the thermoplastic polymer and the properties of the elastomer at the same time. Moreover, PU is extremely resistant to water, oil, heat, fire, ozone, and corrosion [11,12,13,14,15,16,17,18]. Therefore, the demand for using PU is rapidly growing in various applications, such as protective films, shoes, foams, automotive parts, biomedical devices, adhesives, coatings, and so forth [11,12,13,14,15,16,17,18]. Since the DLP method uses polymer resin solutions as printing materials, the DLP method enables the compounding polymers with a variety of fillers to reinforce the properties of polymer matrices [5,11,12]. For instance, PU composites combined with hyaluronic acid and carbon nanotubes (CNTs) were printed three-dimensionally using a DLP-type process [11,12]. If the printed PU sculptures are electrically conductive, it will be possible to expand the application range of PU-based materials for DLP-type 3D printing. Accordingly, it is necessary to develop the electrical properties of PU resin by introducing appropriate fillers.

Conducting polymers (CPs) with conjugated structures are polymeric materials capable of exhibiting electrical conductivity after appropriate doping [13,14,15,19,20,21,22,23,24,25,26,27]. These CPs offer a variety of advantages, including a unique redox behavior, reversible doping/dedoping, processability, they are light-weight and low-cost, and so forth [13,14,15,19,20,21,22,23,24,25,26,27]. Among the various CPs, polyaniline (PANI) provides the widest range of oxidation levels and a comparable or similar level of conductivity to poly(3,4-dioxythiophene) (PEDOT) [13,14,15,21,22,23,24,25,26,27]. In addition, the cost of PANI is about one-hundredth of the price of the PEDOT. For this reason, PANI is regarded as the most cost-effective CP material. Nanoscale PANI, such as nanofibers (NFs) [21,22,23,24,25], nanorods (NRs) [21,22,23], nanoparticles (NPs) [21,22,23], and nanotubes (NTs) [26], are able to form denser conductive paths inside the PU resin because of their improved surface area and electrochemical activity. Thus, PANI is also considered to be among the promising candidates for conductive fillers in 3D printing [25]. The graphene sheet (GS), a two-dimensional (2D) carbon nanomaterial, can provide fascinating properties, such as a high theoretical surface area (2630 m^2^/g), good electrical conductivity due to the high mobility of charge carriers, and high flexibility [16,17,18,24,25,28,29]. These merits of the GS make it a reliable candidate as a filler in PU composites to replace expensive CNTs [16,17,18,28,29]. Despite there being a number of approaches for producing PU composites that employ PANI nanomaterials and GSs, the DLP-type 3D printing of PU/PANI and PU/GS composites is seldom reported [13,14,15,16,17,18]. Thus, it is necessary to study and optimize the conductive 3D printing based on the DLP method using PU/PANI and PU/GS composites.

In this work, the PANI and GS were introduced into PU resins, and DLP-type 3D printing using the PU/PANI and PU/GS composites was studied. Field emission scanning electron microscope (FE-SEM) images were utilized to investigate the presence and dispersion of conductive fillers within the PU resins. The effects of the PANI and GS on the bonding structures and electrical properties of the PU composites were confirmed using Fourier-transform infrared (FT-IR) spectroscopy and a four-point probe measurement, respectively. The printed sculptures of PU/PANI and PU/GS with different filler contents demonstrate high levels of printability and improved electrical properties. Compared to our previous work on the DLP-type 3D printing of polyacrylates, the PU composites prepared in this study exhibit about 4 orders of magnitude enhanced electrical conductivity [25]. Furthermore, the effects of the PANI and GS on the mechanical properties of PU composites were assessed by comparing the stress–strain curves of the 3D-printed products.

## 2. Materials and Methods

Ammonium persulfate (APS, 98%) and aniline (99%) were purchased from Sigma–Aldrich (St. Louis, MO, USA). The GS paste was obtained from MExplorer Co., Ltd. (Ansan, Korea). The GS paste has a density of 25 mg/mL, and the average thickness and lateral size of the GS are about < 5 nm and 2–3 μm, respectively. The flexible PU resin solution (Carima Acryl, CFY063W) was obtained from Carima (Seoul, Korea). Hydrochloric acid (HCl, 35–37%), ethanol (95%), and acetone (99%) were obtained from Daejung Chemical & Metals Co., Ltd. (Siheung, Korea). The PANI NFs used in this experiment were synthesized by a simple chemical oxidative polymerization method [21,22,23,24,25]. A total of 5.5 × 10^−2^ mol of aniline was introduced into 200 mL of 1 M aqueous HCl solution, followed by vigorous stirring for 0.5 h. Then, 2.65 × 10^−2^ mol of APS was added to the reaction medium, reacting at room temperature for 3 h to obtain PANI emeraldine salt (ES) precipitates. As-prepared PANI precipitates were washed with water, ethanol, and acetone solvents.

Preparative conditions of PU composites are summarized in Table 1. The dispersion treatment of the conductive fillers was carried out through vigorous stirring for 5 h at a stirring speed of 600 rpm and a sonication treatment for 0.5 h. The sonication treatments of the conductive fillers were conducted by using an ultrasonic bath (CPX2800H-E, Branson Ultrasonics Co., Danbury, CT, USA) with 110 W power and 40 kHz frequency. To maintain the dispersion temperature at a room temperature, we replaced the cold water in the ultrasonic bath every 0.3 h.

The 3D printer used in this work was a DLP-type printing system (IM-96, Carima, Seoul, Korea). The PU composites, employing different amounts of conductive fillers, were 3D-printed and evaluated for their printability. The maximum printable concentration of the PANI was 6 wt% with respect to the PU solution, and no lamination occurred when the content of PANI exceeded 6 wt%. The maximum printable concentration of the GS was 2 wt% with respect to the PU solution, and no lamination occurred when the GS content exceeded 2 wt%. Images of conductive fillers and 3D-printed sculptures were acquired with a field emission scanning electron microscope (FE-SEM, S-4800, HITACHI, LTD, Tokyo, Japan). The electrical properties of the 3D-printed sculptures were carried out using a 4-point probe conductivity meter (Mode Systems Co., Korea) equipped with a current source meter (Keithley 2400, Keithley Co., Cleveland, OH, USA). The electrical conductivity (σ) measurement formula based on the 4-point probe conductivity method is defined as σ (S/cm) = 1/*ρ* = (ln2/π*t*) (I/V), where *ρ*, *R*, and *t* are the static resistivity, surface resistance, and thickness of samples, respectively [19]. A universal testing machine (UTM, Instron-5543, Instron Co., Norwood, MA, USA) was utilized to measure the mechanical properties following the American Society for Testing and Materials (ASTM) standard D638. The mechanical properties of the samples were recorded with a cross-head speed of 10 mm/min at room temperature under a relative humidity (RH) of 30%.

## 3. Results and Discussion

**Figure 1:**
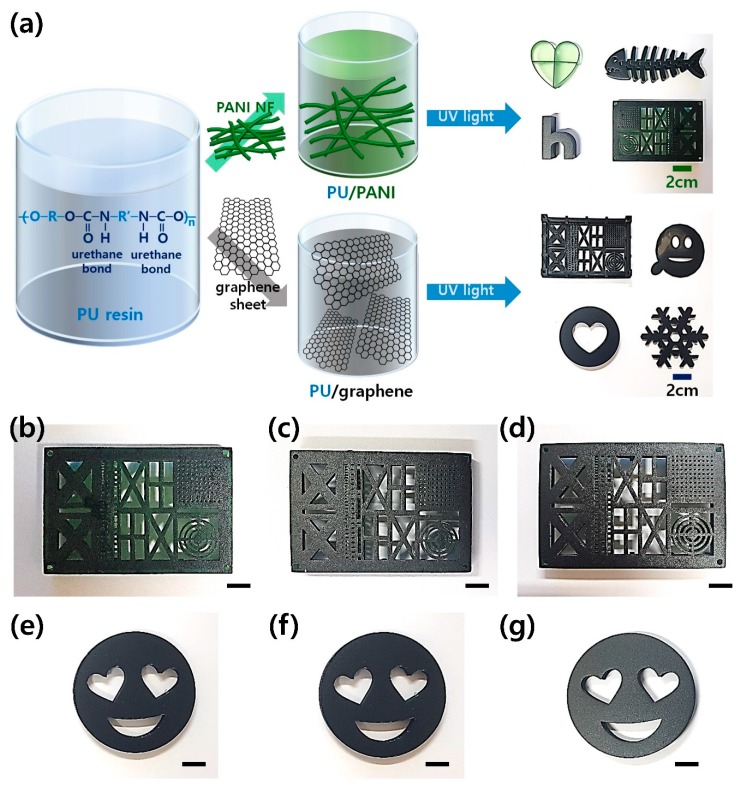
(**a**) Overall process of conductive 3D printing using polyurethane (PU) resin solutions employing a polyaniline (PANI) nanofiber (NF) and a graphene sheet (GS). Digital images of 3D-printed PU composites with different filler contents: (**b**) 1 wt% PANI, (**c**) 3 wt% PANI, (**d**) 6 wt% PANI, (**e**) 0.33 wt% GS, (**f**) 1.00 wt% GS, and (**g**) 2.00 wt% GS (bar size: (**b**) 1 cm, (**c**) 1 cm, (**d**) 1 cm, (**e**) 1 cm, (**f**) 1 cm, and (**g**) 1 cm).

Figure 1a demonstrates the illustration of the DLP-type 3D printing of PU composites. The PANI and GS were dispersed in the PU resin solution, respectively. These conductive fillers were dispersed in the PU resin solution containing a crosslinking agent via mechanical stirring and ultrasonic treatments. The role of the PANI and GS is to form conjugated paths for electron delocalizations within the PU resin, resulting in highly conductive PU sculptures even after the 3D printing [13,14,15,16,17,18,24,25,28,29]. The PU resin serves as a dispersion medium for the conductive fillers, and the PU matrix enables the desired size and shape of sculptures after the DLP-type 3D printing [25]. After the 3D printing under a UV light with a wavelength of about 300 nm is done, conductive PU sculptures of various shapes and sizes can be easily obtained by photocrosslinking between the PU prepolymers [11,12]. The colors of printed sculptures filled with the PANI and GS are dark green and dark gray, respectively. As shown in the digital images of the 3D-printed sculptures, it is evident that the PU sculptures embedded with different amounts of PANI and GS were printed successfully (Figure 1b–g). This suggests that both the PANI and GS were highly dispersible with the PU resin at various filler contents. In our experimental conditions, the maximum concentrations of the PANI and GS for the 3D printing of PU composites were 6 wt% and 2 wt%, respectively. This indicates that the printability of PU resins is highly affected by filler contents.

**Figure 2:**
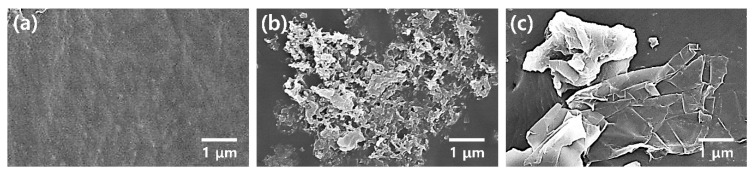
Field emission scanning electron microscope (FE-SEM) images of 3D-printed PU composites with different fillers: (**a**) pristine PU, (**b**) PU/PANI with 6 wt% PANI, and (**c**) PU/GS with 2 wt% GS.

Figure 2 shows FE-SEM images of pristine PU, PU/PANI, and PU/GS composites. There is no filler in the FE-SEM image of the pristine PU resin, suggesting that the PU serves as a matrix of the PANI and GS (Figure 2a). In the FE-SEM image of the PU composite filled with 6 wt% of PANI nanomaterials, the diameters and lengths of PANI nanomaterials were 40–60 nm and 0.2–0.4 μm, respectively (Figure 2b). The length of the PANI shown in Figure 2a is shorter than the initial length (0.6–1.5 μm) of the PANI NFs shown in Figure S1a (see Supplementary Materials). This indicates that the PU resin plays a role in reducing the size and length of PANI NFs [22,23]. In addition, continuous ultrasound may destroy the PANI chains, which would result in smaller sizes of the PANI NFs [30]. 

**Figure 3:**
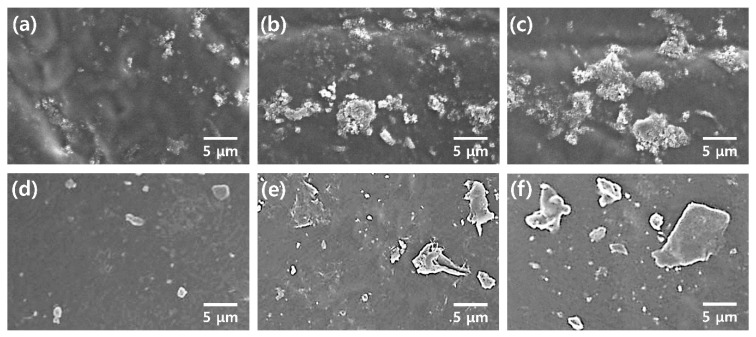
FE-SEM images of 3D-printed PU composites with different filler contents: PU/PANI with (**a**) 1 wt% PANI, (**b**) 3 wt% PANI, and (**c**) 6 wt% PANI. PU/GS with (**d**) 0.33 wt% GS, (**e**) 1 wt% GS, and (**f**) 2 wt% GS.

FE-SEM images of the PU composites filled with different amounts of PANI are shown in Figure 3a–c. The area of PANI embedded in the PU matrix becomes larger with increasing PANI content, and the intermaterial aggregations also increase with the filler content. The maximum printable concentration of PANI in the PU resin was 6 wt%, and no lamination occurred when the content of PANI exceeded 6 wt%. The aggregation of PANI nanomaterials is related to strong hydrogen bonding, dipole–dipole, and London forces between the PANI chains [21,22,23,24]. The sizes of the graphene sheets ranged from 2 to 5 μm and were found in the PU/GS composite (Figure 3d–f). The Raman spectrum of the GS used in this work shows peaks at 1349, 1575, and 2661 cm^−1^, corresponding to the D band, G band, and 2D band, respectively (Figure S1b, see Supplementary Materials) [16,17,31]. The D and G bands refer to the breathing mode and the first-order scattering of the *E*_2g_ vibrational mode of *sp*^2^ carbon atoms, respectively. A broad 2D band at around 2661 cm^–1^ is indicative of the few-layered GSs. In addition, the intensity ratio of the D band to the G band (*I*_D_/*I*_G_) of the GS used in this work is about 0.39, and the *I*_D_/*I*_G_ value is about a quarter of the reduced graphene oxides (RGOs) reported by previous studies [16,17,31]. These results suggest that the GS used in this work is different from the RGO. In the FE-SEM images of PU composites filled with different amounts of GS, more GSs were found to be more widespread at higher contents of GS (Figure 3d–f). The increased size of GS clusters is ascribed to van der Waal’s interactions, such as dipole–dipole and London forces, between each GS [16,17,18,24,25,28,29]. Considering these results, the PANI and GS expand conductive channels within the PU matrix, while the aggregation of PANI and GS disturbs the crosslinking between the PU prepolymers [32]. Therefore, the maximum loading amounts of the PANI and GS in the 3D printable PU composites were fixed at 6 and 2 wt%, respectively.

**Figure 4:**
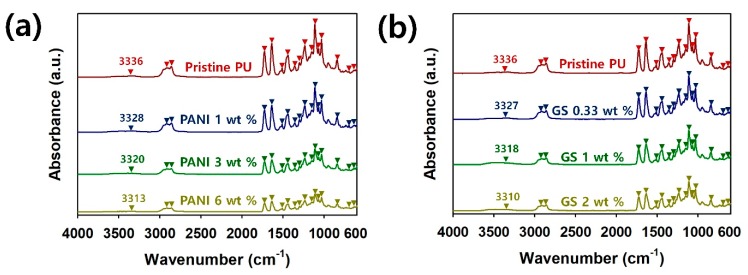
Fourier-transform infrared (FT-IR) spectra of 3D-printed PU composites with different filler contents: (**a**) PU/PANI and (**b**) PU/GS.

To confirm the bond structures of the 3D-printed PU sculptures, the Fourier-transform infrared (FT-IR) spectra of pristine PU, PU/PANI, and PU/GS composites are shown in Figure 4. The characteristic peaks for the PU are found at the following wavenumbers: 697, 729, 841, 953, 1032, 1088, 1112, 1140, 1235, 1359, 1442, 1510, 1636, 1726, 2862, 2918, and 3336 cm^–1^ (Table S1, see Supplementary Materials) [16,31,33,34]. In the PU composites filled with conductive fillers, the characteristic peaks for the PANI and GS were not found. However, it is evident that the absorbance of the PU peaks is reduced with increasing amounts of PANI and GS, indicating that both the PANI and GS were successfully introduced into the PU matrix. Interestingly, the corresponding peak for N–H stretching in the pristine PU shifts to a lower wavelength with increasing filler contents. These blue shifts are found in every FT-IR spectrum of PU/PANI and PU/GS composites (Figure 4a,b). The results indicate that the hydrogen bonding interactions between PU chains are weakened by both PANI and GSs [34]. Therefore, the interaction forces of the PU with the conductive fillers are enhanced to improve the processability of the PU composites.

**Figure 5:**
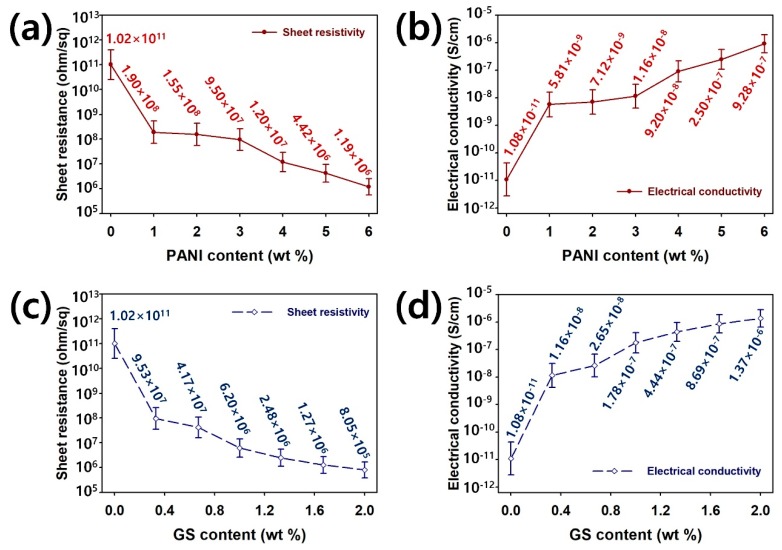
(**a**) Sheet resistance and (**b**) electrical conductivity of 3D-printed PU/PANI composites with different PANI contents; (**c**) Sheet resistance and (**d**) electrical conductivity of 3D-printed PU/GS composites with different GS contents.

Figure 5 summarizes the electrical properties of PU composites filled with the PANI and GS. The sheet resistivity of the PU/PANI composites decreases with increasing PANI content, while the electrical conductivity of the PU/PANI composites increases with the filler content (Figure 5a,b). Sheet resistance and electrical conductivity of the PU/PANI containing 6 wt% of PANI are 8.57 × 10^4^ times lower (1.19 × 10^6^ ohm/sq) and 8.57 × 10^4^ times higher (9.28 × 10^−7^ S/cm), respectively, compared with the pristine PU (1.02 × 10^11^ ohm/sq and 1.08 × 10^–11^ S/cm). This indicates that conductive channels for electron delocalization in the PU are successfully created by introducing PANI nanomaterials [13−15,24,25]. At a higher content of GS, the PU composites demonstrate a lower sheet resistance and a higher electrical conductivity than the PU (Figure 5c,d). The PU/GS composite with 2 wt% of GS exhibits 1.27 × 10^5^ times lower (8.05 × 10^5^ ohm/sq) and 1.27 × 10^5^ times higher (1.37 × 10^−6^ S/cm), respectively, compared with the pristine PU (1.02 × 10^11^ ohm/sq and 1.08 × 10^–11^ S/cm). Considering these results, it is assumed that the GS offers more conductivity improvement compared with the PANI [16,17,18,24,25].

**Figure 6:**
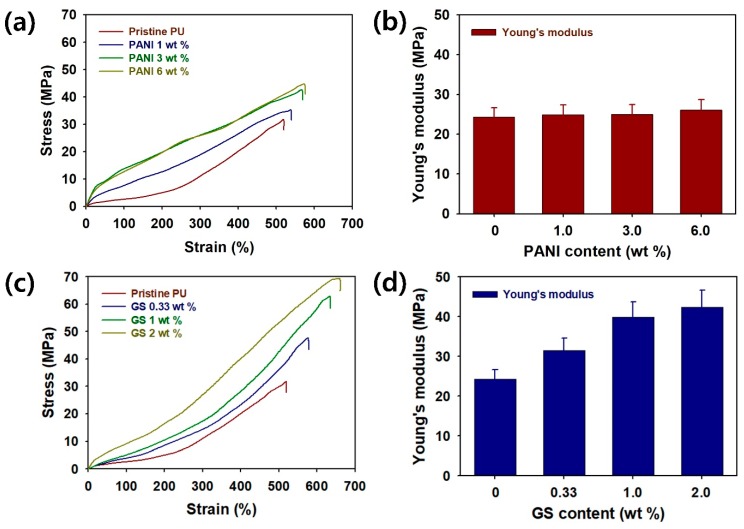
(**a**) Stress–strain curves and (**b**) Young’s modulus values of 3D-printed PU/PANI composites with different PANI contents; (**c**) Stress–strain curves and (**d**) Young’s modulus values of 3D-printed PU/GS composites with different GS contents. Young’s modulus (*E*, MPa) was calculated according to an equation *E* = stress (MPa)/strain (%).

To evaluate the practical applicability of the 3D-printed sculptures, stress–strain curves, and Young’s modulus values of the PU filled with PANI and a GS are shown in Figure 6a–d. The tensile strength (MPa) of the pristine PU and PANI composites, including 1 wt% PANI, 3 wt% PANI, and 6 wt% PANI, are 31.6, 35.0, 42.5, and 44.5, respectively (Figure 6a). In addition, the elongation at the break point (%) of PU/PANI composites was found to increase in the following order: pristine PU (5.20 × 10^2^) < 1 wt% PANI (5.40 × 10^2^) < 3 wt% PANI (5.70 × 10^2^) < 6 wt% PANI (5.76 × 10^2^) (Figure 6a). Young’s modulus (MPa) of the PU/PANI composites increases in the following order: pristine PU (24.3) < 1 wt% PANI (24.9) < 3 wt% PANI (25.0) < 6 wt% PANI (26.1) (Figure 6b). These results suggest that the PANI nanomaterials are effective in reinforcing the flexibility and toughness of PU/PANI composites. Although both the tensile strength and Young’s modulus of the PU/PANI composites increase with increasing filler contents, the differences between pristine PU and PU/PANI composites are not significant. This suggests that the formation of the PANI clusters retards the reinforcing effects on the ultimate strength of the PU/PANI composites [35]. When the GS was added into the PU matrices, it was clear that both the tensile strength and elongation at the break point of PU/GS composites were significantly improved. The tensile strength (MPa) of the pristine PU/GS composites increases in the following order: pristine PU (31.6) < 0.33 wt% (47.6) < 1 wt% (62.6) < 2 wt% (69.3) (Figure 6c). Furthermore, the elongation at the break point (%) of the PU/GS composites increases in the following order: pristine PU (5.20 × 10^2^) < 0.33 wt% (5.79 × 10^2^) < 1 wt% (6.36 × 10^2^) < 2 wt% (6.61 × 10^2^). These results indicate that the GS was a suitable filler to achieve tougher and stronger PU sculptures after the DLP-type 3D printing. Such improvements in PU/GS composites are attributable to the intrinsic advantages of the GS, such as robustness, mechanical strength, and flexibility [36]. Moreover, Young’s modulus (MPa) of PU/GS composites increases as follows: pristine PU (24.3) < 0.33 wt% (31.5) < 1 wt% (39.8) < 2 wt% (42.4) (Figure 6d). Overall, it was clear that the GS provides better reinforcing effects as a filler of PU resins compared with the PANI.

## 4. Conclusions

In this comparative study, the DLP-type 3D printing of PU/PANI and PU/GS composites with different filler contents was investigated. The conductive PU composites prepared by our work were able to be printed as conductive sculptures with different sizes and shapes. Furthermore, the presence of PANI and a GS in the PU resin matrices was proven using FE-SEM images and FT-IR spectra. In the FE-SEM images of the 3D-printed PU sculptures, conductive fillers were observed to be widespread within the PU matrices. The optimal amounts of PANI and GS in the 3D printable PU composites were 6 wt% and 2 wt%, respectively. FT-IR spectra of PU composites demonstrate the reduced absorbance for PU composites after the introduction of the PANI and GS, and the presence of either the PANI or GS weakens the interactions between the PU prepolymers. The PU/PANI composite employing 6 wt% of PANI exhibits an 8.57 × 10^4^ times lower sheet resistance value compared with the pristine PU. After a proper amount of GS (2 wt% with respect to the PU resin solution) was introduced, a significant decrease in the sheet resistance of 1.27 × 10^5^ times occurred. Both the PANI and GS provide conductive channels within the PU matrices even after the 3D printing is completed. The sheet resistance and electrical conductivity obtained from the PU/PANI and PU/GS sculptures are sufficient to demonstrate the antistatic properties of the PU composites. Considering that the minimally required sheet resistance for antistatic agents and conducting pastes are 10^11^ Ω/sq and 10^7^ Ω/sq, the conductivity values obtained from our work are sufficient to ensure high-performances for both antistatic and conducting materials [37]. In addition, as both the thermoelectric (TE) figure of merit and the power factor (PF) of the materials are dependent on electrical conductivity, our work will be applicable to the thermoelectric (TE) materials [38]. The stress–strain curves of the PU composites reconfirm that the GS and PANI are able to effectively improve the mechanical properties of the PU. Thus, our work on the DLP-type 3D printing of PU/PANI and PU/GS composites will further accelerate the application of conductive PU sculptures for a variety of applications, such as antistatic materials, conducting pastes, TE materials, automotive parts, heat dissipation pads, sensor, electrochemical, optical, biomedical devices, and so forth.

## Figures and Tables

**Table 1:** Preparative conditions of PU composite resin solutions.

Sample	PU Resin (g)	PANI NFs (g)	GS (g)
pristine	20.00	-	-
1 wt% PANI	19.80	0.2	-
2 wt% PANI	19.60	0.4	-
3 wt% PANI	19.40	0.6	-
4 wt% PANI	19.20	0.8	-
5 wt% PANI	19.00	1.0	-
6 wt% PANI	18.00	1.2	-
0.33 wt% GS	19.93	-	0.067
0.67 wt% GS	19.87	-	0.13
1.00 wt% GS	19.80	-	0.20
1.33 wt% GS	19.74	-	0.26
1.67 wt% GS	19.67	-	0.33
2.00 wt% GS	19.60	-	0.40

## Supplementary Materials

The following are available online at https://www.mdpi.com/2073-4360/12/1/67/s1, Figure S1: (a) FE-SEM image of polyaniline nanofibers (PANI NFs) and (b) Raman spectrum of graphene sheet (GS) used in this work. Table S1: Characteristic bands with specific vibrational modes of PU composites.
