# Supplementary material for: Comparative Studies on Polyurethane Composites Filled with Polyaniline and Graphene for DLP-Type 3D Printing
Source: Polymers (Basel). 2020 Jan 2;12(1):67. doi: 10.3390/polym12010067 (PMC7023528; doi:10.3390/polym12010067)
Supplement: Supplementary file 1 [file polymers-12-00067-s001.pdf]

Supplementary Materials for:

polymers

Comparative studies on Polyurethane Composites filled with Polyaniline and Graphene for DLP-type 3D Printing

Hyeonseo Joo [1] and Sunghun Cho [1,*]

[1] School of Chemical Engineering, Yeungnam University, Gyeongsan 38541, Republic of Korea.

*E-mail: shcho83@ynu.ac.kr
Tel.: +82-53-810-2535

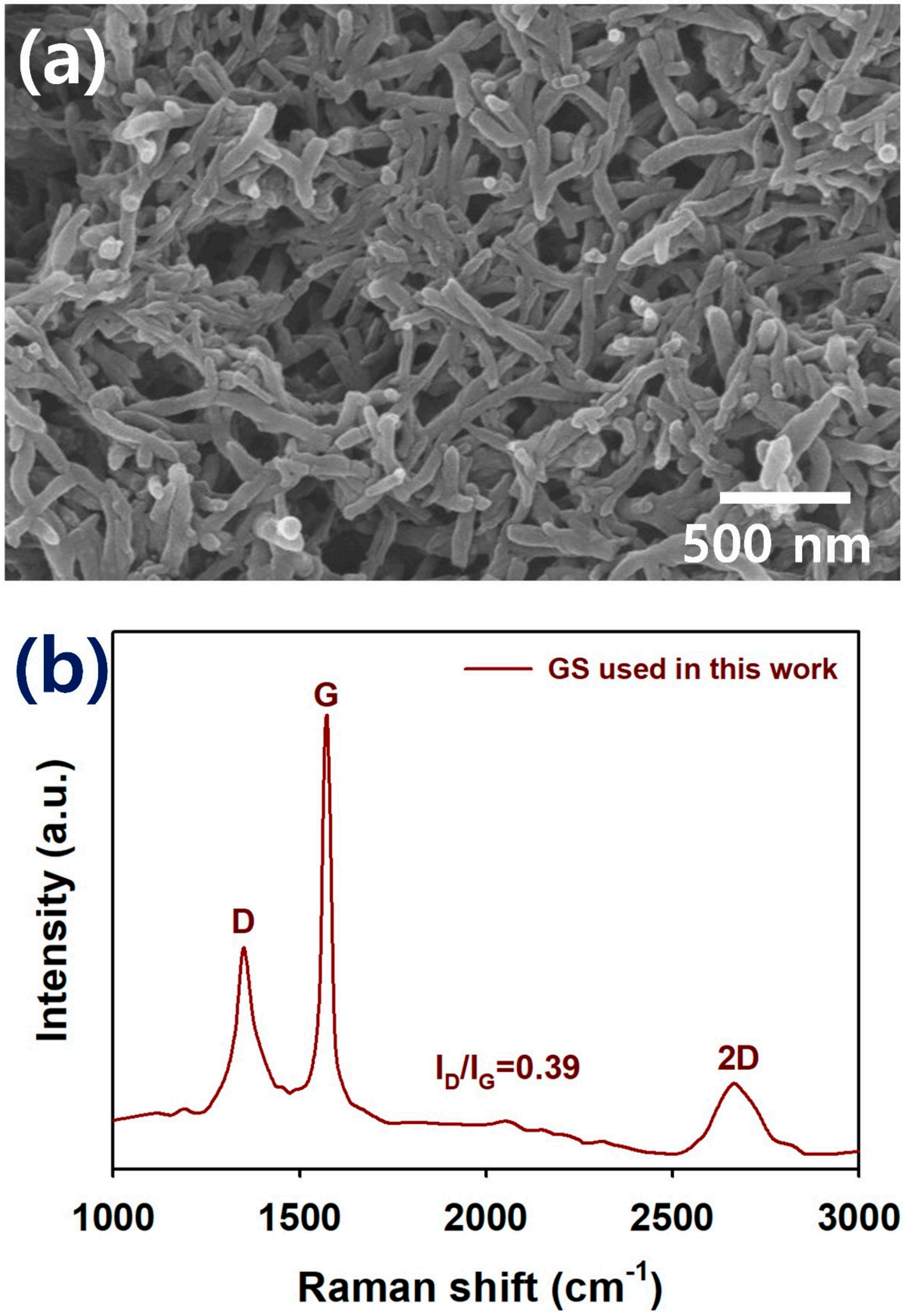

Figure S1. (a) FE-SEM image of polyaniline nanofibers (PANI NFs) and (b) Raman spectrum of graphene sheet (GS) used in this work.

Table S1. Characteristic bands with specific vibrational modes of PU composites.

Wavenumber (cm^{-1})	Vibration
697, 729	C=C out-of-plane bending
841, 953	C–H out-of-plane bending
1032, 1088	C–O–C symmetric stretching
1112	C–O–C asymmetric stretching
1140	C–H in-plane bending
1235,1359,1442,1510	C–N stretching, N–H stretching
1636	C=C stretching
1726	C=O stretching
2862	C–H symmetric stretching
2918	C–H asymmetric stretching
3310–3336	N–H stretching of secondary amine
